# Atypical Vascular Involvement in a Case of Behçet's Disease

**DOI:** 10.1155/2012/848101

**Published:** 2012-11-28

**Authors:** Alejandro Rodríguez Morata, Ana Hidalgo Conde, Carlos de la Cruz Cosme, Susana Gómez Ramírez, Rafael Gómez Medialdea

**Affiliations:** ^1^Department of Angiology and Vascular Surgery, Hospital Virgen de la Victoria, Campus de Teatinos s/n, 29010 Málaga, Spain; ^2^Department of Internal Medicine, Hospital Virgen de la Victoria, Campus de Teatinos s/n, 29010 Málaga, Spain; ^3^Department of Neurology, Hospital Virgen de la Victoria, Campus de Teatinos s/n, 29010 Málaga, Spain

## Abstract

*Introduction*. Behçet's disease (BD) is a form of vasculitis of unknown etiology which is rare in our environment. It is characterized by a variety of clinical manifestations and usually affects young adults. Recurrent oral and genital ulcers are a characteristic and extremely frequent symptom, but mortality is linked with more significant symptoms such as aortic pseudoaneurysm, pulmonary pseudoaneurysm, and cerebral venous thrombosis. *Patient and Method*. We present a case of a young male with atypical BD and severe polyvascular involvement (previous cerebral venous thrombosis and current peripheral venous thrombosis, acute ischemia, and peripheral arterial pseudoaneurysm) who required urgent surgical intervention due to a symptomatic external iliac pseudoaneurysm. *Result*. The pseudoaneurysm was successfully treated, we performed an iliofemoral bypass, and we treated it with steroids and immunosuppressive therapy. *Conclusions*. These rare clinical manifestations highlight the importance of considering BD in young patients, even in usual cases of vascular intervention, whether arterial or venous in nature.

## 1. Introduction

Behçet's disease (BD) is a systemic vasculitis of unknown etiology with a low prevalence in our society [1]. It is theorized that infectious pathogens trigger systemic inflammatory responses if certain genetic predispositions are present (the disease is associated with HLA-B51, which is up to 4 times more prevalent in patients with BD than in control subjects). However, none of the theories are conclusive [2].It mainly affects young adults (predominantly male) and the diagnosis is essentially clinical. BD can be found with different manifestations of severity and can affect virtually any organ. The most common manifestations take the form of recurrent oral or genital ulcers and other skin conditions. However, less frequent but potentially fatal complications could be developed, such as central or peripheral arterial lesions, in the form of saccular pseudoaneurysm [3] with a high risk of rupture.

## 2. Case Presentation

The patient was a 35-year-old man—who operated a jackhammer. He appeared at the Emergency and Accidents Department with constant and intense pain in the lower left limb, moderate ipsilateral paresis in the ankle, foot, and toes, and coldness. He had suffered these symptoms for around 48 hours. In addition, he had suffered from an irregular fever (not measured using a thermometer) for two weeks with oropharyngeal discomfort and spontaneous pain in the groin and left lower quadrant which increased with time. He did not mention any previous trauma but when pressed recalled hyperpulsatility beneath the inguinal ligament at least a year before. He did not consult a physician as the problem did not appear to bother him. 

His medical history included a cerebral venous thrombosis (left transverse and sigmoid sinus thrombosis) which was attributed to mastoiditis in 2006, resulting in occasional attacks of epilepsy being treated successfully with oxcarbazepine. He was a smoker with no known allergies.

A physical examination revealed feverishness, a generally good physical condition, normal thorax and abdomen features, and an expansive throbbing mass below the inguinal ligament and left minor pelvis. The absence of femoral and infrainguinal pulses was obvious as well as severe and acute ischemic symptoms. The other limbs were found normal.

**Figure 1:**
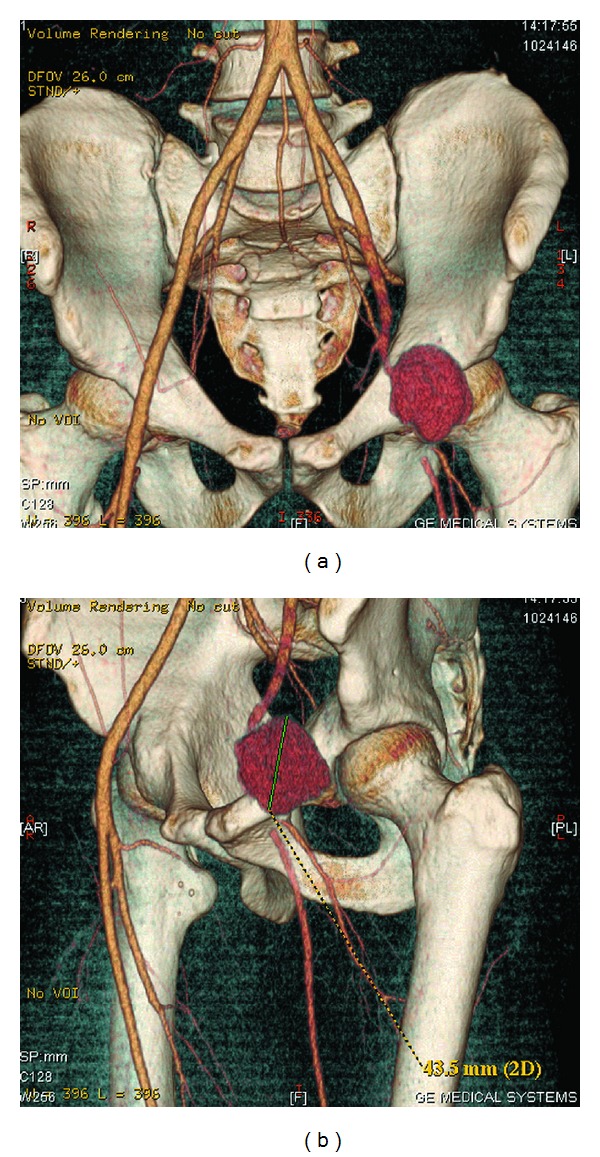
CT angiography: pseudoaneurysm of the external iliac artery with acute arterial thrombosis in the common femoral artery bifurcation.

A blood test discovered leukocytosis (16 × 10^3^/microliter) and neutrophilia (*N* = 80.4%) with a hemoglobin value of 13 g/dL. In a CT scan we observed a saccular aneurysm with a transversal diameter of 45 mm located in the left external iliac artery with no sign of rupture. This was associated with a short occlusion in the bifurcation between the femoral and profunda arteries (Figure 1). A deep venous thrombosis in the iliofemoral area was found. There was no evidence of aneurysms or malformations in other abdominal organs. 

The patient underwent urgent surgery under general anesthesia. During the surgery, we decided first to perform a left retroperitoneal approach in order to prepare field for the clamping of the iliac artery, above the aneurysmatic lesion. Then, we used a longitudinal approach in the left groin to dissect the common femoral, femoral, and profunda arteries, preparing for the clamping.

**Figure 2:**
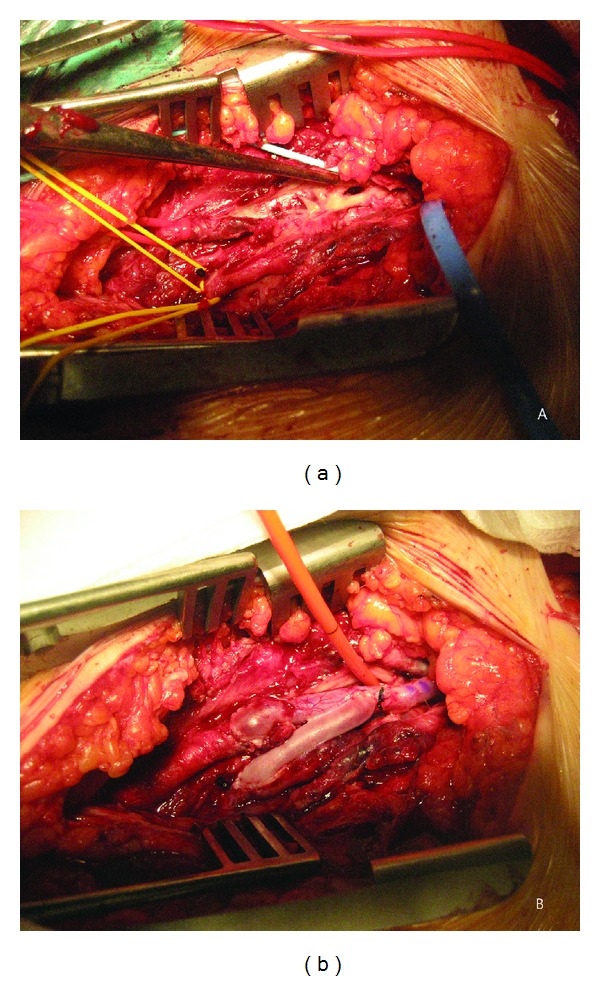
(a) Femoral and profunda arteries being clamped from the inguinal approach, with vessel loop. The retroperitoneal approach shows a vessel loop check of the external iliac artery. The tweezers point directly to the neck of the pseudoaneurysm. A vessel loop crosses the bypass tunnel. (b) Sequential inverted saphenous vein bypass joining the external iliac and femoral and profunda arteries in the patient's left leg. The rigid probe monitors the neck of the pseudoaneurysm, properly excluded.

The dissection was made with difficulty due to advanced fibrosis. With full heparinization we clamped the external iliac, femoral, and profunda arteries. We opened the anterior wall of the pseudoaneurysmatic mass. There was not any mural thrombosis and it had the appearance of a common traumatic pseudoaneurysm. We performed a thrombectomy with a Fogarty catheter and removed the short thrombus formed in the common femoral bifurcation. Neither pus nor any friable tissue was found macroscopically to support an infectious etiology. We easily observed the neck of the pseudoaneurysm, with an endothelized appearance (Figure 2(a)) suggesting a traumatic etiology. However, the patient could not recall any direct trauma previously. We took specimens from the common femoral artery around the neck and from the wall of the pseudoaneurysm, for further culture and pathological studies.

A revascularization of the femoral and profunda arteries was performed using a sequential inverted bypass originated in the healthy part of the external iliac artery. We used the saphenous vein of the contralateral thigh, prepared for this purpose (Figure 2(b)). The pseudoaneurysmatic capsule was not removed due to the high level of morbidity associated with this action, its adherence to adjacent tissues, and the absence of any local signs of sepsis. 

In the immediate postoperative period, the patient recovered his distal pulses and the pain and paresis of the limb disappeared. However,the patient did experience significant edema of the limb, difficulties in venous return, and reperfusion. A Doppler scan found a deep venous thrombosis from the iliac to the popliteal veins.

During the early stages of postoperative distress,the patient had a fever of up to 39°C with no evident site of infection and was unsuccessfully treated with piperacillin/tazobactam. The microbiology results were negative for blood, urine, bronchial aspirate, mural thrombus, and tonsillar thrush exudate. Serology was negative for hepatotropic viruses, HIV, and syphilis. The postoperative blood analysis showed some changes, which are presented in Table 1 for the purpose of clarity. The pathological examination of the artery only showed unspecific fibrous thickening and loss of elastic and muscle fibers. The examination of the capsule confirmed the nature of the pseudoaneurysm, without the integrity of the vessels wall, showing mainly fibrous tissue. The microbial culture of the specimen was negative.

The persistence of an irregular fever of up to 38.5°C with no clear focus, oral aphthous lesions, massive venous thrombosis, previous cerebral venous thrombosis, and pseudoaneurysm supported a diagnosis of BD. The pathergy test was negative, but although this test is very specific it is known to have low sensitivity and excessive geographical variability in our environment [1]. In addition, the patient recalled having an ulcerated scrotal lesion which was by then a jagged scar of about 1 cm. 

Following the diagnosis of BD, an extensive examination was carried out. On examining the eyes, we ruled out a retinal vasculitis. When screening for other vascular lesions, CT angiography of the skull, thorax, abdomen, and pelvis revealed no abnormalities. Thrombophilia and antibody screening proved negative, there were no common tumor markers, and the transthoracic echocardiography showed no pathological signs.

The patient was treated with Prednisone at doses of 1 mg/kg/day with a very good response. The fever disappeared within 24 hours, as did the oral thrush and acute phase reactants. This dose was reduced progressively as much as we could and this treatment was continued during 22 months. The patient was also treated with anticoagulant therapy (Acenocumarol, INR: 2,5) during 15 months (until we could check the complete recanalization with ultrasound) and he wore an elastic high-compression stocking. He was treated with immunosuppressive drugs (Azathioprine 150 mgr/24 h) and Colchicine (1 mgr/24 h) too, and both drugs are his treatment actually, with lower dose of Azathioprine (100 mgrc/24 h). The outcome at two years shows a normal duplex scan in the bypass, a complete recanalization with venous insufficiency in femoropopliteal venous system, and a complete clinical remission of his vasculitis.

## 3. Discussion 

We present this case of BD with vascular involvement because we believe it offers three areas of special interest. 

First is the difficulty of an accurate early diagnosis with no florid symptomatology and, in particular, when there is a possible diagnosis that is potentially fatal and requires urgent surgical intervention, as in the case of a possible mycotic or traumatic pseudoaneurysm in the iliac artery. 

Secondly, we find this case especially interesting because of its unusual vascular involvement. In this patient, his BD most probably began with his neurovascular episode (dural venous sinus thrombosis) three years before the current episode. The location is the most unusual thrombotic location (9%) of all thromboses affecting the venous territory and is certainly the most serious [4]. Furthermore, the arterial complications are the least frequent (4.1%) of all the possibilities involved in BD and, of these complications, the most common sites are the abdominal aorta and pulmonary arteries, not the peripheral arteries, which are in fact the rarest [3–5]. 

Thirdly, the semiology presented at the start of the case history and subsequent events are of interest. The patient's history started with an episode of Neuro-Behçet's (dural venous thrombosis and epilepsy) and continued through a latent period culminating in this actual episode. The inguinal hyperpulsatility occurred two years after the initial event. It is known that up to 32% of patients with BD can experience phases of remission [2] but we cannot exclude the possibility that the period of latency was, in fact, characterized by lesions which were not found because the patient did not consult a physician. Equally, the patient had suffered from a sore throat at times, this having been attributed to tonsillitis. In all probability it was a recurrent oral aphthosis. It is striking that both the Neuro-Behçet's and the vascular disease entities tend to occur later (5–10 years following the onset of the disease) [6].

The involvement of the vascular tree in BD is a common phenomenon although not with arterial involvement but in the form of deep vein thrombosis (up to 85% of patients) [7]. In the review of the literature we have established that thrombosis can involve the superficial and deep veins of the lower and upper extremity but may also involve central large veins, especially the superior and inferior vena cava. Less common manifestations include hepatic vein thrombosis causing Budd-Chiari syndrome, mesenteric vein thrombosis, renal vein thrombosis, and intracranial thrombosis involving the dural sinus [8]. Pulmonary artery thrombosis and intracardiac thrombus have also been reported [9]. 

Both occlusive arterial disease and aneurysm formation occur as manifestations of Behçet's disease, but arterial aneurysms occur more frequently than occlusive disease. The incidence may be less than 5% [10]. Arterial disease usually manifests 3 to 8 years after the initial diagnosis, at a mean age of 30 years [11]. The abdominal aorta is the most common site of aneurysm formation, followed in decreasing order by the pulmonary, femoral, popliteal, brachial, and iliac arteries. Rare cases involving the carotid, vertebral, coronary, and visceral arteries have been reported [12–15].

In the review of the literature we found higher percentage of thrombophilic abnormalities in patients with BD than in patients with thrombosis without vasculitis. Thus, mutations of Factor V Leiden have been found in up to 37%, mutations of the prothrombin 20210A in up to 31%, and elevated anticardiolipin IgG antibodies in up to 30% of cases [8]. In our patient, the thrombophilia screening was negative despite the occurrence of cerebral venous thrombosis and peripheral venous and arterial thrombosis.

In this case, we considered a saphenous vein graft to be the best option for the revascularization of the limb and the exclusion of the pseudoaneurysm in this patient, considering the preoperative diagnosis of mycotic pseudoaneurysm. Notwithstanding, if we have been aware that his symptoms were caused by BD, we would have chosen the same graft anyway. They are known for their excellent results [16] in the endovascular exclusion of the aneurysm, though we know the outcome of a stent under the inguinal ligament is usually its long-term fracture and secondary thrombosis.

In conclusion, we successfully treated the patient by means of a medical and surgical method in this case of BD with atypical features. This unusual clinical presentation emphasizes the importance of considering this disease in cases like this and, of course, in any unusual cases of vascular arterial and venous events.

## Figures and Tables

**Table 1:** Relevant analytical data postoperatively.

Hemogram	Biochemistry	Electrophoresis and C-reactive protein	Autoimmunity	Thrombophilia	TM markers
L.: 16.300 *μ*L	ALT: 101 UI/L	CRP: 145 mgr/L	Anti-DNA: (−)	AT III (−)	*α*FP: (−)
N.: 13.105 *μ*L	GGT: 280 UI/L	*α*_1_: 0.65 g/dL	ANCA: (−)	prot. C y S (−)	CEA: (−)
Hb.: 10.1 gr/dL	Fe: 17 mgr/dL	*α*_2_: 1.40 g/dL	AntiB2 GPI: (−)	RPCa (−)	CA19.9: (−)
Pl.: 585000 *μ*L	TF: 134 mgr/dL Ferritin: 584 mgr/dL	*β*_2_: 0.49 g/dL	LA1: (−) ANCA: (−) ASLO: (−) RF: (−)	Anticardiolipin (−) V Leyden F. (−)	CYFRA: (−) PSA: (−)

L: leukocytes; N: neutrophil; Hb.: hemoglobin; Pl: platelet; ALT: alanine aminotransferase; GGT: gamma-glutamyltranspeptidase; TF: transferring; RF: rheumatoid factor; AT III: antithrombin 3.
